# Supplementary figures and images for: Identification of immune-related signatures and pathogenesis differences between thoracic aortic aneurysm patients with bicuspid versus tricuspid valves via weighted gene co-expression network analysis
Source: PLoS One. 2023 Oct 26;18(10):e0292673. doi: 10.1371/journal.pone.0292673 (PMC10602290; doi:10.1371/journal.pone.0292673)

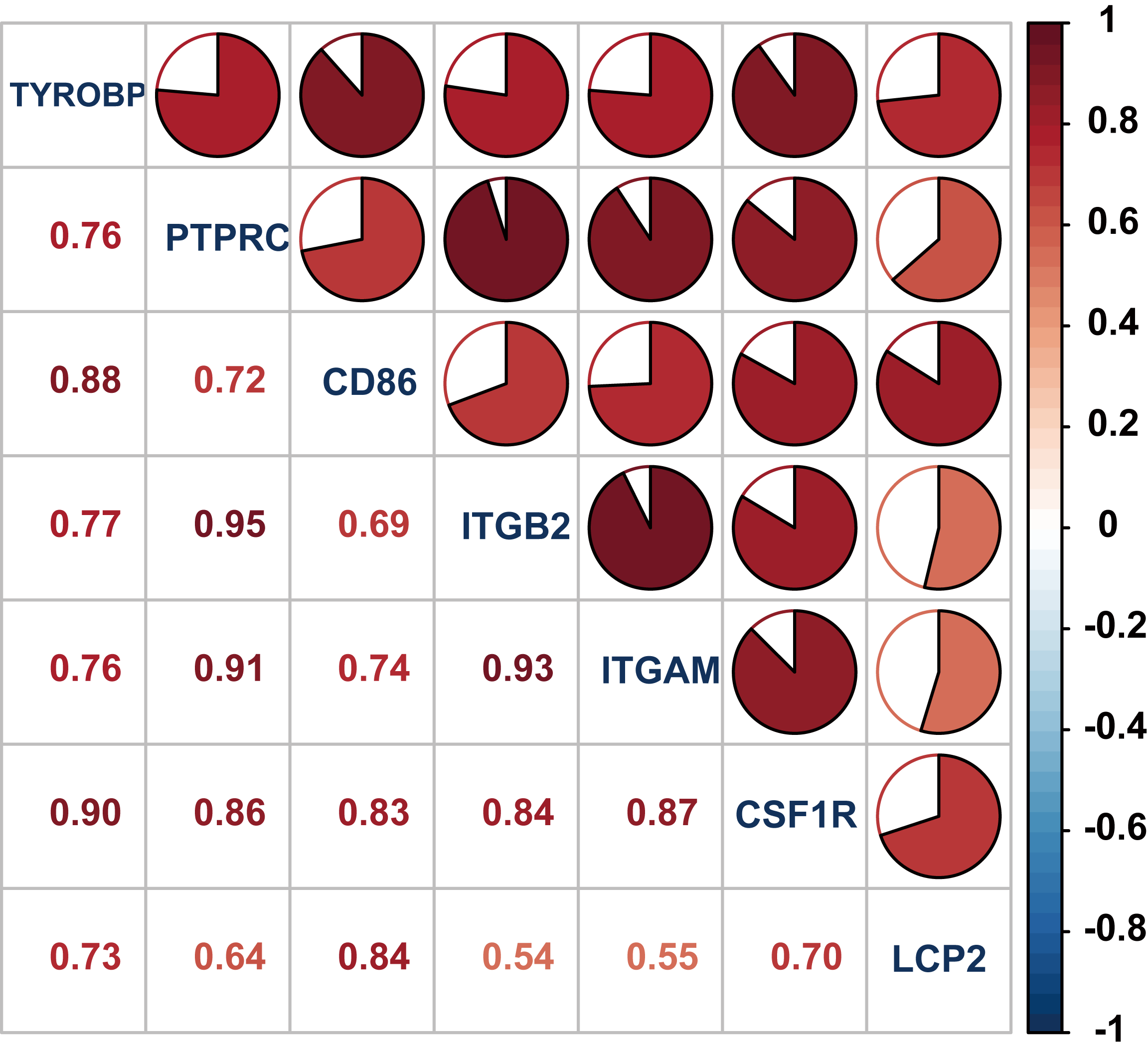

Supplement: S1 Fig — Shared between both top-10 degree and betweenness centrality measurement lists, when compared to each other. (TIF) [file pone.0292673.s001.tif]

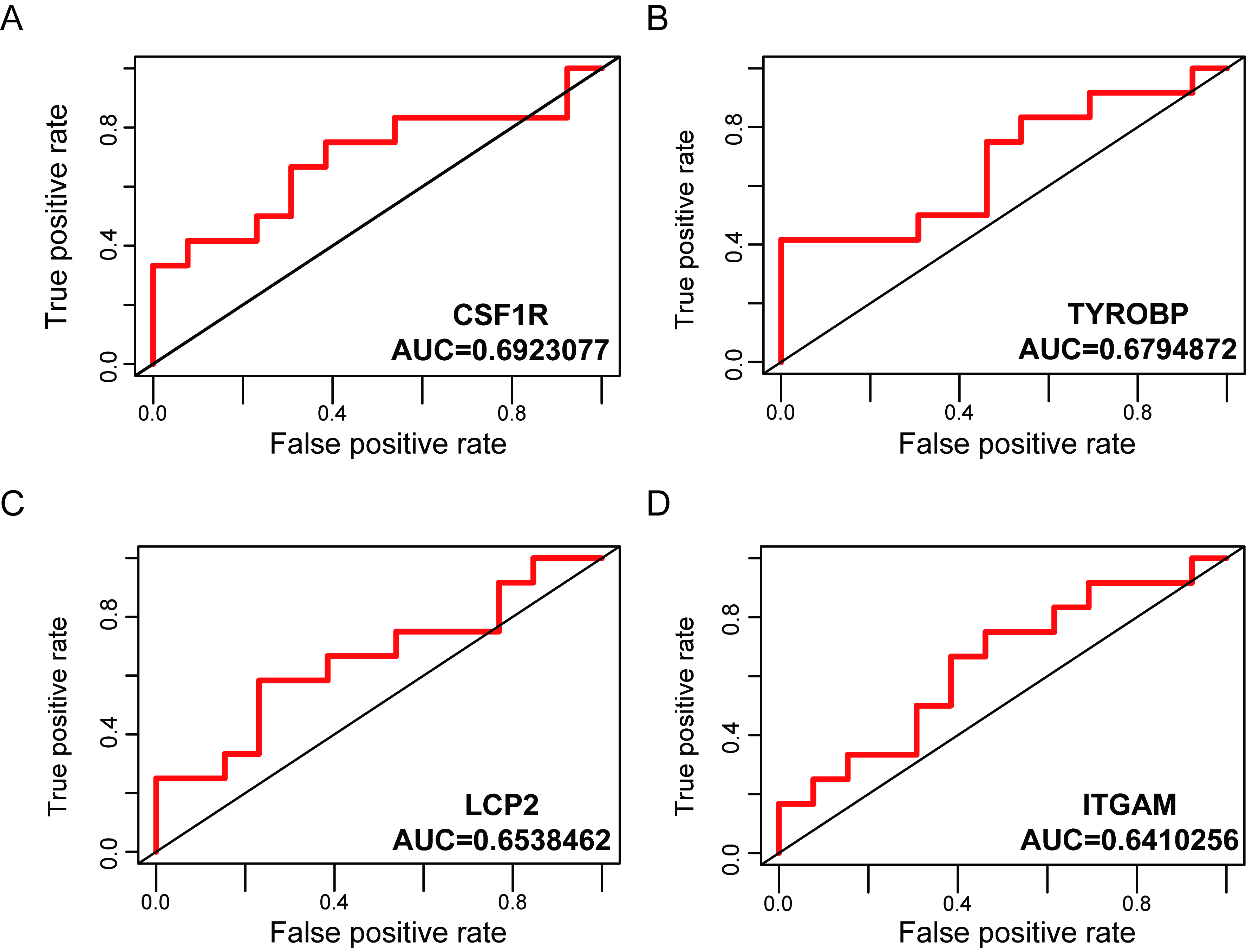

Supplement: S2 Fig — (A) Colony stimulating factor 1 receptor (CSF1R). (B) TYRO protein tyrosine kinase binding protein (TYROBP). (C) Lymphocyte cytosolic protein 2 (LCP2). (D) Integrin alpha M (ITGAM). (TIF) [file pone.0292673.s002.tif]
